# Higher rating of perceived exertion and lower perceived recovery following a graded exercise test during menses compared to non-bleeding days in untrained females

**DOI:** 10.3389/fphys.2023.1297242

**Published:** 2024-01-11

**Authors:** Morgan Delp, Grant A. Chesbro, Brian A. Pribble, Ryan M. Miller, Hugo M. Pereira, Christopher D. Black, Rebecca D. Larson

**Affiliations:** ^1^ Body Composition and Human Performance Lab, Department of Health and Exercise Science, University of Oklahoma, Norman, OK, United States; ^2^ Neuromuscular Research Lab, Department of Health and Exercise Science, University of Oklahoma, Norman, OK, United States; ^3^ Human Movement and Neurophysiology Lab, Department of Health and Exercise Science, University of Oklahoma, Norman, OK, United States; ^4^ Sensory and Muscle Function Lab, Department of Health and Exercise Science, University of Oklahoma, Norman, OK, United States

**Keywords:** rating of perceived exertion, perceived recovery, menstrual cycle, VO_2max_, sex differences

## Abstract

The underrepresentation of the female population in exercise sciences could be attributed, at least in part, to difficulty in appropriately accounting for the effects of the menstrual cycle (MC). Previous studies examining the effects of the MC on aerobic performance and subjective measures of aerobic performance show conflicting results.

**Purpose:** The study examined how the MC affects the objective and subjective measures of aerobic performance within untrained female participants and in comparison with untrained male participants assessed at similar time intervals.

**Methods:** Twenty-one participants (12 females and 9 males) completed a graded exercise test (GXT) on a cycle ergometer. The female participants were tested during their early follicular (EF; menses), ovulatory (O), and mid-luteal (ML) phases of the MC. The male participants were included as the control group and were randomly assigned to a menstrual cycle phase for each visit. During GXT, maximal oxygen consumption (VO_2max_), respiratory exchange ratio (RER), maximal heart rate (HR_max_), peak blood lactate, and rating of perceived exertion (RPE) were determined. Twenty-four hours post-exercise, the perceived recovery status (PRS) was assessed. The MC phase was estimated using basal body temperature (BBT) in the female participants.

**Results:** The male participants obtained a higher peak power and VO_2max_ compared to the female participants (*p* < 0.05). All objective measures of aerobic performance did not significantly differ across the MC phases or time points that were tested. In the untrained female participants, an effect of the MC phase on RPE was found, with RPE being higher at EF (8.92 ± 0.79) compared to O (7.67 ± 1.23; *p* < 0.05) and ML (7.75 ± 1.06; *p* < 0.05). In addition, an effect of the MC phase on PRS was found, with perceived recovery being lower at EF (6.83 ± 0.94) compared to O (8.83 ± 1.12) and ML (8.67 ± 0.65; all *p* < 0.005) for the untrained female participants. No significant differences in RPE and PRS were found between tests in the untrained male participants. The female participants had lower perceived recovery following EF (6.83 ± 0.94) compared with the male participants (9.00 ± 1.00; *p* < 0.001).

**Conclusion:** The untrained female participants perceived greater exertion during GXT and impaired recovery following GXT in EF compared to O and ML. These results may be attributed to either a drop in female sex hormone concentrations or discomfort associated with menses. The male participants did not exhibit any changes over time. Future studies using subjective parameters such as perceived exertion to track the internal load of training in the naturally menstruating female population should consider menses.

## 1 Introduction

In exercise sciences, sex is a biological variable that researchers often control to reduce group variability. Inadvertently, however, this has led to the female population being largely underrepresented in exercise physiology research (Oosthuyse and Bosch, 2010). The average participation of the female population in exercise science articles was estimated to be in the range of 35%–37% (Costello et al., 2014). The underrepresentation of the female population in exercise science research could be attributed, at least in part, to difficulty in appropriately accounting for the effects of the menstrual cycle. In the last few decades, the prevalence of the female population in athletic activities and recreational exercise has increased (Acosta and Carpenter, 1977; Smith and Wrynn, 2013), motivating researchers to advance their current understandings of how the menstrual cycle (MC) and the changes in estrogen and progesterone potentially impact physiological and perceptual responses to exercise (Jurkowski et al., 1981; Hackney et al., 1991; Cook et al., 1998; Constantini et al., 2005).

Maximal oxygen consumption (VO_2max_) is an indicator of cardiorespiratory fitness and is a predictor of all-cause and disease-specific mortality (Harber et al., 2017). Research on the effect of the MC on VO_2max_ has shown conflicting results. A previous study with physically active participants found that VO_2max_ was affected by the menstrual cycle (Gordon et al., 2018). Using an incremental stress test on a cycle ergometer, the study found that there was a difference in VO_2max_ (mL/kg/min) between the menses phase (days 1–3; 41.6 ± 3.7), mid-follicular phase (days 9–11; 44.1 ± 3.9 v. 44), and pre-menstrual phase (days 27–28; 43.2 ± 3.6) (Gordon et al., 2018). In contrast, other studies have observed that VO_2max_ assessed using an incremental stress test is not affected by the MC phase in female participants (Dombovy et al., 1987; Bemben et al., 1995; Smekal et al., 2007; Taipale-Mikkonen et al., 2021). The participants of the studies were reported as moderately active to trained (Bemben et al., 1995; Smekal et al., 2007), while one study had untrained participants (Dombovy et al., 1987).

However, several of these studies did not report perceptual variables such as rating of perceived exertion (RPE) or perceived recovery status (PRS) (Bemben et al., 1995; Smekal et al., 2007; Gordon et al., 2018). In moderately active or trained female participants, the MC did not affect perceived exertion measured during exercise and perceived exertion at maximal effort (Nicklas et al., 1989; De Souza et al., 1990; Bailey et al., 2000; Lara et al., 2020; Pereira et al., 2020). In contrast, the influence of the MC on perceived exertion shows conflicting results in untrained individuals (Dombovy et al., 1987; Nicklas et al., 1989; Pivarnik et al., 1992; Galliven et al., 1997; Caldwell Hooper et al., 2011). These studies employed a variety of methods for determining the MC phase, including blood samples (Nicklas et al., 1989; Pivarnik et al., 1992) or based on their menstrual history or calendar(Dombovy et al., 1987; Caldwell Hooper et al., 2011), and a variety of exercise intensities, including submaximal (Nicklas et al., 1989; Pivarnik et al., 1992; Caldwell Hooper et al., 2011) and maximal (Dombovy et al., 1987). Two studies found no change in RPE across the phases (Dombovy et al., 1987; Nicklas et al., 1989), while the other two studies found that RPE was affected by the MC phase (Pivarnik et al., 1992; Caldwell Hooper et al., 2011). One study found that RPE was higher during the luteal phase than the follicular phase during a 60-min bout of sustained cycling (Pivarnik et al., 1992). Another study found that RPE was higher during the early follicular phase than the late follicular and luteal phases during sustained cycling (Caldwell Hooper et al., 2011). The differences found in the studies could be due to methodological differences in how the menstrual cycle phases were determined and whether subtle disturbances (small hormone fluctuations, inadequate levels of hormones, etc.) in the menstrual cycle may have affected the perception of performance (Janse De Jonge et al., 2019). In addition, the influence of the training status on the menstrual cycle may have affected the performance and perceived performance of the participants (Janse De Jonge et al., 2019). The differences between the findings in trained and untrained individuals could simply be due to the training status, given that sedentary individuals and individuals with low fitness levels have been shown to have heightened RPE at a given heart rate (HR) compared to trained individuals (Demello et al., 1987; Travlos and Marisi, 1996; Chen et al., 2002).

The inconsistent findings of the effects of the MC on the perception of effort are potentially supported by the interaction of gonadal hormones with nociception in the peripheral and central nervous system (Fillingim and Ness, 2000). Estrogen and progesterone have been suggested to influence the magnitude of pain perception which is a key determinant of perceived exertion and recovery (Craft et al., 2004; Martin, 2009). Pain perception is likely integrated into RPE, and a correlation between heightened pain and heightened RPE during exercise has been observed (Black and Dobson, 2013). In addition, there are conflicting observations in the reported subjective measures of pain sensitivity across the menstrual cycle in female participants—with some finding these values to be the lowest during the early follicular phase, ovulation, or pre-menstrual phase (Iacovides et al., 2015). Meanwhile, several other studies have reported no significant changes in pain sensitivity across the menstrual cycle (Choi et al., 2006; Teepker et al., 2010; Iacovides et al., 2015).

Understanding how perceived exertion during exercise and perceived recovery following exercise are affected by the menstrual cycle could have implications for training. It has been suggested that knowing how people perceive their efforts may be as important as knowing how much effort they are exerting (Rejeski, 1981). RPE allows for the assessment of an individual’s perception of effort during performance. In addition, RPE can be used as a method for assessing training load when training and has been shown to be an accurate method compared to HR-based methods (Borresen and Lambert, 2008). The differences in how exercise is perceived at different points during the menstrual cycle may have an impact on how trainers or individuals approach training loads.

Improving the prescription of exercise in training programs for untrained, naturally menstruating female participants depends on an adequate understanding of any potential influence of the MC phase on both objective and subjective measures of aerobic performance. This may have implications for how personal trainers or other exercise professionals approach training with currently untrained female participants who are not using hormonal contraceptives. If VO_2max_ varies during the menstrual cycle, then scheduling exercise testing could be affected. In addition, if the perception of effort varies across the MC, then this would affect how trainers approach prescribing training loads and/or how they might motivate their clients. The purpose of this study was to examine how the MC affects the subjective and objective variables of aerobic performance within the female participants and in comparison with the male participants at similar time intervals.

## 2 Methods and materials

### 2.1 Participants

This study includes results from 21 participants, comprising 9 males (age: 21.6 years ±1.7, height: 177.4 cm ± 2.8, and weight: 82.9 kg ± 4.8) and 12 females (age: 21.3 years ±1.1, height: 167.9 cm ± 1.7, and weight: 71.0 kg ± 4.6). The male participants were treated as the control group in this experiment due to the absence of fluctuations in the levels of progesterone and estradiol (Barbieri, 2014). An *a priori* sample size calculation indicated a minimum of 20 individuals for a two-group (males vs. females), three-timepoint (menstrual cycle phases) study to indicate differences using a within–between repeated measures ANOVA with an α value of 0.05, effect size of 0.30, and power of 0.80 (Lebrun et al., 1995; Gordon et al., 2018). Additionally, in this study, male and female participants were required to be free from external hormonal influences (hormonal contraceptives, antidepressants, or other medications) and were required to maintain the current physical activity levels for the duration of the study. The female participants were required to have a regular menstrual cycle while not using oral hormonal contraceptives, intrauterine devices, implants, injections, or any other hormonal birth control methods for at least 1 year. All female participants reported a regular cycle lasting ≥21 days and ≤35 days (cycle length: 29.33 days ±3.30) and a luteal phase of ≥10 days based on ovulation detected using basal body temperature (BBT) measurements. Participants were free from metabolic, respiratory, neurological, and cardiovascular diseases, and free from any debilitating musculoskeletal injury (within 1 year). In addition, none of the participants were engaged in a regular training regimen, which was defined as not meeting ACSM recommendations for physical activity. All participants provided written informed consent before participating in the study. This study was approved by the local Institutional Review Board and complied with the Declaration of Helsinki.

### 2.2 Design

This study utilized a repeated measures design, across three timepoints—during menses (day 0–3), which was considered the early follicular phase (EF), the ovulation phase (O), and the mid-luteal phase (ML; between 7 and 9 days following the spike in BBT)—with similar time intervals between visits for the male participants. The participants completed a Profile of Mood States (POMS) at all three visits. In addition, the female participants received a menstrual cycle history questionnaire. The first visit included a familiarization with the GXT protocol and equipment. Following the familiarization trial, visits were scheduled to control for the time of day (within a 2-h start range). The participants were instructed to refrain from vigorous exercise for 48 h and fast 3 h prior to data collection and were also advised not to consume any caffeine within 6 h prior to testing.

### 2.3 Menstrual cycle tracking

The female participants were scheduled for visits during menses as an indication of an early follicular phase (EF; within the first 3 days of menstruation, day 0–3), ovulatory (O; within 24 h of a spike in BBT ≥ 0.3°C), and mid-luteal (ML; 7–9 days following the spike in BBT) phases. BBT was tracked in all participants using a digital thermometer (iProvèn Model BBT-113Ai, Beaverton, OR, USA). BBT was measured immediately after waking to ensure the most accurate reading. Participants were required to track and report BBT each morning for 4 weeks before and throughout the study. This established a baseline of BBT values for each individual and aided in the confirmation of ovulation (McClure Brown, 1973). Further visits were scheduled according to which phase occurred first following the familiarization visit. This ensured that the visit order was randomized across female participants, which served to minimize the influence of the learning effect on the test results. For the female participants, four participants had their first visit during EF, two participants had their first visit during O, and six participants had their first visit during L. The time between visits varied between subjects due to differences in female participants’ cycle and availability (Barbieri, 2014). Visit dates for male subjects were randomly assigned to correspond to the time between visits for the average female 28-day cycle. For example, if a male participant was assigned a visit equivalent to EF first, his visit equivalent to O occurred approximately 14 days after, and his visit equivalent to ML was approximately 7–9 days later. The male participants were randomly assigned to visit on analogous days according to a predicted 28-day cycle. For the male participants, three had their first visit scheduled as “EF,” four had their first visit scheduled as “O,” and two participants started testing in “ML.”

### 2.4 Graded exercise test protocol

The GXT protocol was performed on a cycle ergometer (Lode Excalibur Sport, The Netherlands). Participants were fitted with a Polar heart rate sensor (Polar Inc. Model H1, Bethpage, NY, USA) to track the heart rate. Before GXT, participants performed a 5-min warm-up at a self-selected intensity. GXT consisted of a ramp protocol starting at 0.5 W/kg of body weight and increasing by 0.5 W/kg every 60 s (Larson et al., 2015). The participants were required to maintain a self-selected cycling cadence; if the cadence declined more than 10 rpm, then the test was terminated. Following the cessation of the test, the participants performed a cooldown at 25 W until their heart rate dropped below 130 bpm.

Expired gases were collected and analyzed using a metabolic cart (ParvoMedics TrueOne 2400, Sandy, UT, USA). VO_2max_ was recorded breath-by-breath continuously during the exercise test using a 30-s average. During GXT, RPE and lactate were measured at the end of each minute of exercise. At the end of the test, the last stage HR (HR_max_) respiratory exchange ratio (RER), and peak power (power achieved during the last stage) were recorded.

### 2.5 VO_2max_ verification

To ensure a true maximal value for oxygen consumption, the participants rested for 20 min before performing a verification test on the cycle ergometer. The load of the verification test was set at the peak power of the previously administered GXT. The verification test began with a 10-s warm-up at 0.5 W per kilogram of the participant’s body weight and increased every 5 s, ensuring that the participants reached the max load within 30 s of cycling. If the participant maintained a cadence of at least 40 rpm for the majority of the 5 min, or if the participant’s VO_2_ value exceeded the previous measure (±3%) (Nolan et al., 2014), then the participant’s previous maximal graded exercise test was deemed invalid. If these criteria were not met and the participant stopped cycling or cycled at a cadence <40 rpm within 5 min, then the original maximal test was considered valid, and all measured parameters were recorded. All of the participants met the criteria for a valid test. Cooldown was set at 25 W, and subjects were free to dismount the bike when their HR measured ≤130 bpm and they felt comfortable walking and exiting the laboratory safely.

### 2.6 Blood lactate

The blood lactate level was assessed by performing a finger stick drawing of ∼1.0 μL blood; samples were analyzed using a portable lactate analyzer (Lactate Plus, Nova Biomedical, Waltham, MA, USA). Blood lactate was collected before and immediately after the GXT.

### 2.7 Rating of perceived exertion

The participants rated their exertion based on a modified Borg scale ranging from 0 (no exertion at all) to 10 (maximal exertion) (Borg, 1982). They were instructed to point to the number corresponding to their *overall* RPE.

### 2.8 Perceived recovery status

Twenty-four hours after each visit, the participants were asked to complete a perceived recovery status (PRS) scale, which ranged from 0 (very poorly recovered and extremely tired) to 10 (very well recovered and highly energetic) (Laurent et al., 2011).

### 2.9 Profile of Mood States

A shortened version of the POMS questionnaire was used for the study (Shacham, 1983). The questionnaire consists of a series of descriptions that describe the feelings that the participants may have. The participants self-reported on each of these areas for how they were feeling on the day of each test. The scoring ranged from 0 to 4, with 0 being “not at all” and 4 being “extremely.” The categories of interest were dealing with feelings of fatigue and vigor on the day of each test. POMS was used as a way to examine if there were changes in moods over the menstrual cycle for the female participants and over time in the male participants.

### 2.10 Statistical analysis

Statistical analysis for this study was performed using IBM SPSS Statistics version 28 (IBM Corp., Armonk, NY, USA). A two-group (female and male) by two-condition (GXT and verification) by three-timepoint (MC phase/time point) repeated measures ANOVA was used to examine differences in VO_2max_. A two-group (female and male) by three-timepoint (MC phase/time point) repeated measures ANOVA was performed for the objective (HR_max_, RER, and blood lactate) and subjective measures (overall RPE, local RPE, and PRS). Pairwise comparisons were performed using a Bonferroni correction. The effect size for each variable was calculated using partial eta-squared (η_p_^2^), in which small effect = 0.01–0.05, medium effect = 0.06–0.13, and a large effect size ≥ 0.14 (Cohen, 1988).

Intraclass correlation coefficients (ICCs) and their 95% confidence intervals for the objective parameters (VO_2max_, RER, HR_max_, and lactate) were calculated based on a mean-rating (k = 3), absolute agreement, two-way mixed effects model (Koo and Li, 2016). ICCs were interpreted as follows: values less than 0.50 indicated poor reliability, values between 0.50 and 0.75 indicated moderate reliability, values between 0.75 and 0.90 indicated good reliability, and values greater than 0.90 indicated excellent reliability (Koo and Li, 2016).

## 3 Results

### 3.1 Objective measures

There were no observed main effects of the MC phase for the female participants or main effects of time for the male participants on the objective measures (VO_2max_, RER, HR_max_, or peak blood lactate) (Table 1). In addition, there were no interactions between MC phase/time and sex for any of the objective measures (Table 1). There were no observed differences within sex for VO_2max_ recorded from GXT and verification protocols (Table 1). There was an effect of sex on VO_2max_ (F_1,19_ = 318.896, *p* < 0.001, ηp^2^ = 0.944) and peak power (F_1,19_ = 548.377, *p* < 0.001, ηp^2^ = 0.967), where the male participants had a higher VO_2max_ (mean difference: 9.44 mL kg^-1^ ∙ min^-1^; *p* = 0.026, ηp^2^ = 0.236) and higher peak power (mean difference: 86.96 W; *p* < 0.001, ηp^2^ = 0.472) than the female participants (Table 1). The objective measures were observed to have moderate to excellent test–retest reliability (Table 2).

**TABLE 1:** Aerobic parameters.

	Early follicular (EF)	Ovulation (O)	Mid-luteal (ML)
VO_2max_ (ml ∙ kg^-1^ ∙ min^-1^)			
*Females*	29.59 ± 6.26*	30.23 ± 6.80*	30.42 ± 6.95*
*Males*	39.77 ± 11.64	38.99 ± 10.57	39.79 ± 11.68
Verification VO_2max_ (ml ∙ kg^-1^ ∙ min^-1^)			
*Females*	29.39 ± 6.45	28.73 ± 6.49	28.89 ± 6.64
*Males*	36.76 ± 11.02	36.14 ± 10.11	37.16 ± 10.25
HR_max_ (BPM)			
*Females*	175.96 ± 9.04	179.63 ± 7.18	179.42 ± 7.63
*Males*	175.56 ± 12.88	172.89 ± 8.29	170.06 ± 6.34
RER			
*Females*	1.23 ± 0.08	1.26 ± 0.07	1.22 ± 0.09
*Males*	1.23 ± 0.08	1.23 ± 0.05	1.23 ± 0.05
Lactate (mmol ∙ L^-1^)			
*Females*	9.41 ± 1.66	9.88 ± 1.56	10.13 ± 2.17
*Males*	10.92 ± 1.72	10.43 ± 1.42	10.80 ± 1.27
Peak power (watts)			
*Females*	202.42 ± 40.68*	206.67 ± 38.27*	201.25 ± 41.41*
*Males*	288.78 ± 65.90	290.00 ± 54.77	292.44 ± 56.82

Values are represented as mean ± SD; RER, respiratory exchange ratio; * indicates differences from male participants; *p* < 0.05.

**Table 2:** Test–retest reliability.

	ICC	95% CI
VO_2max_		
*Group*	0.922	0.984–0.997
*Male*	0.993	0.978–0.998
*Female*	0.985	0.961–0.995
Verification VO_2max_		
*Group*	0.928	0.859–0.968
*Female*	0.872	0.703–0.957
*Male*	0.940	0.830–0.985
RER		
*Group*	0.718	0.426–0.876
*Male*	0.415	0.192–0.915
*Female*	0.804	0.502–0.938
HR_max_		
*Group*	0.774	0.538–0.900
*Male*	0.662	0.047–0.914
*Female*	0.843	0.599–0.950
Lactate		
*Group*	0.817	0.622–0.920
*Male*	0.797	0.370–0.950
*Female*	0.811	0.514–0.940
RPE		
*Group*	0.541	0.106–0.792
*Male*	0.574	0.499–0.900
*Female*	0.570	0.028–0.854
PRS		
*Group*	0.657	0.266–0.852
*Male*	0.905	0.701–0.977
*Female*	0.488	0.061–0.822

RER, respiratory exchange ratio; HR_max_, maximal heart rate; RPE, rating of perceived exertion; PRS, perceived recovery scale.

### 3.2 Subjective measures

**FIGURE 1:**
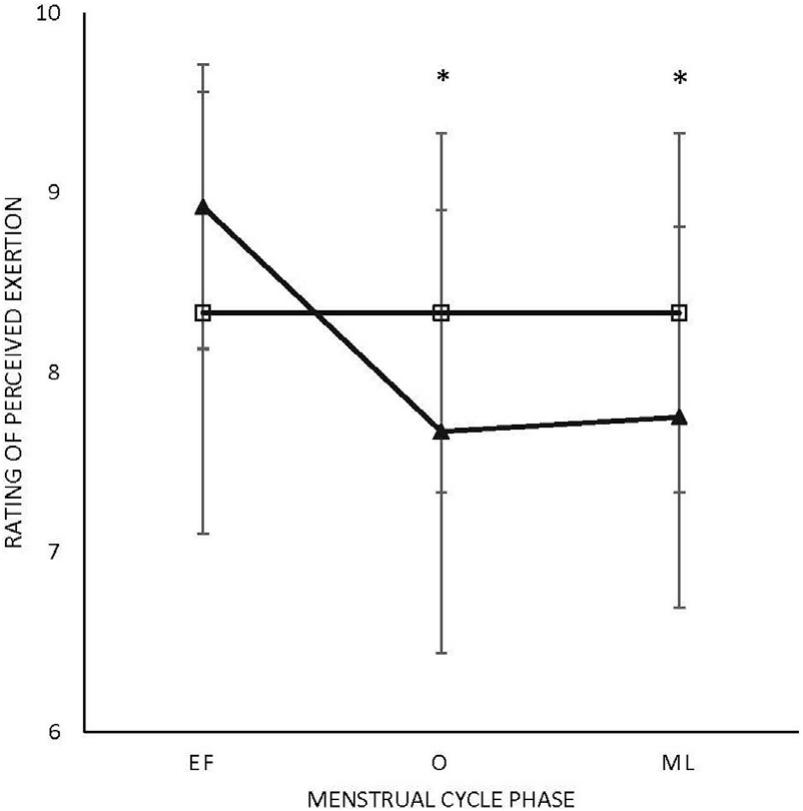
Rating of perceived exertion. Triangle: female participants; box: male participants.*Difference in menses for female participants; EF: early follicular; O: ovulatory; ML: mid-luteal (*p* < 0.05). All values are represented as mean + SD.

**FIGURE 2:**
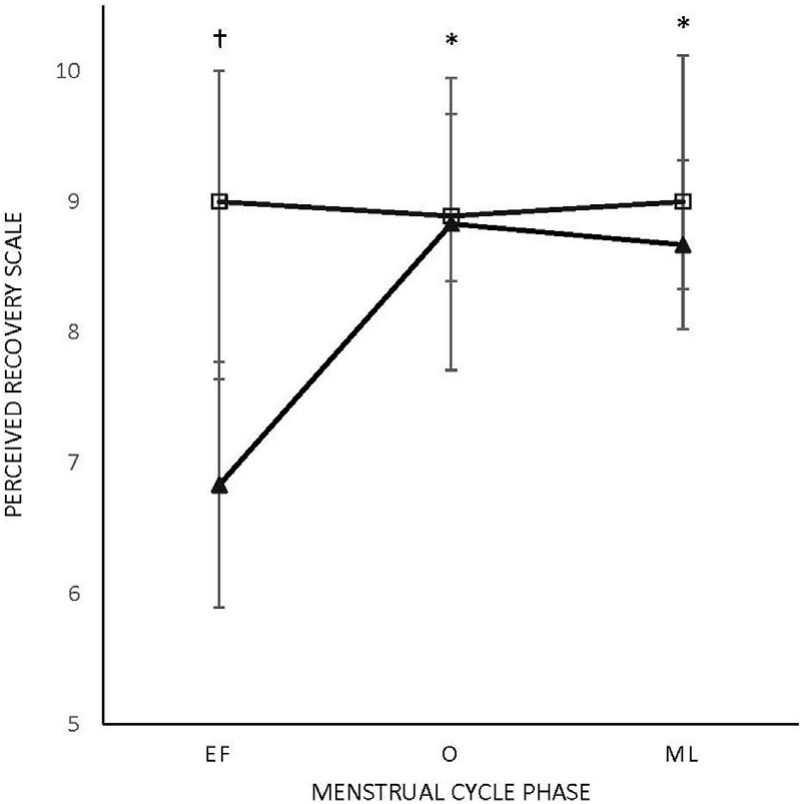
Perceived recovery scale (PRS).Triangle: female participants; box: male participants; M: menses; O: ovulatory; L: luteal. * Difference in menses for female participants. †Difference from male participants (*p* < 0.005). All values are represented as mean + SD.

**FIGURE 3:**
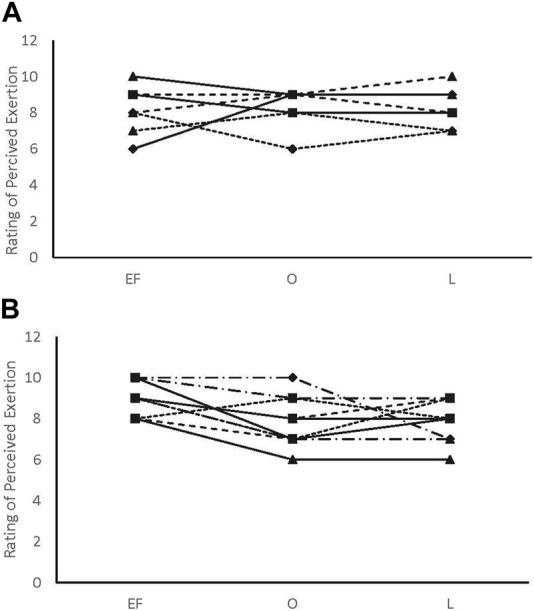
Rating of perceived exertion individual data. **(A)** male participants; **(B)** female participants.

**FIGURE 4:**
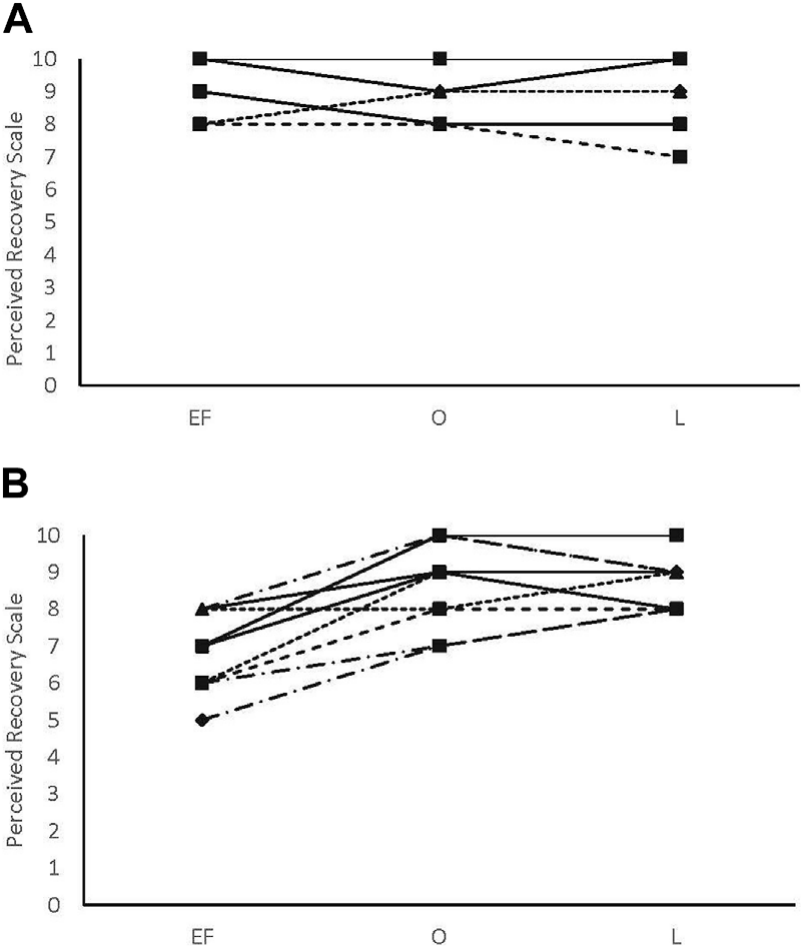
Perceived recovery score individual data. **(A)** male participants; **(B)** female participants.

There were no main effects or interactions for any of the POMS categories for either sex. There was a phase-by-sex interaction for overall RPE (F_2,38_ = 3.543, *p* = 0.039, ηp^2^ = 0.157). Overall RPE was higher at EF (8.92 ± 0.79; ηp^2^ = 0.416) than O (7.67 ± 1.23; *p* = 0.008) and ML (7.75 ± 1.06; *p* = 0.013) for the female participants (Figure 1). A medium effect of sex at each timepoint (EF: 0.09, O: 0.09, and L: 0.08) was observed, with the male participants reporting lower RPE at EF and higher RPE at O and L compared to the female participants (Figure 1). There was a phase-by-sex interaction for perceived recovery (F_2,38_ = 21.972, *p* < 0.001, ηp^2^ = 0.536). Perceived recovery was lower (i.e., they felt less recovered) following EF (6.83 ± 0.94; ηp^2^ = 0.825) compared to O (8.83 ± 1.12; *p* < 0.001) and ML (8.67 ± 0.65; *p* < 0.001) for the female participants (Figure 2). The female participants (6.83 ± 0.94) had lower perceived recovery after GXT during EF than the male participants (9.00 ± 1.00; *p* < 0.001, ηp^2^ = 0.577) (Figure 2). There were no observed differences between visits for the subjective parameters for the male participants (Figure 1; Figure 2). Individual data for RPE are shown in Figure 3, and individual data for PRS are shown in Figure 4.

## 4 Discussion

The purpose of this study was to examine how the MC affects the subjective and objective variables of aerobic performance within the female participants and in comparison with the male participants over similar time intervals. There were no differences in the objective measures over the phases of the menstrual cycle assessed in the study in the female participants, but there were differences in the subjective measures (Table 1). Specifically, perceived exertion was higher during EF (8.92 ± 0.79) than O (7.67 ± 1.23) and ML (7.75 ± 1.06) in the female participants, while in the male participants, there was no effect of time (Figure 1). Perceived recovery was lower in EF (6.83 ± 0.94) than O (8.83 ± 1.12) and ML (8.67 ± 0.65) in the untrained female participants, while the same effect was not seen in the untrained male participants (Figure 2). In addition, perceived recovery was lower during EF in the female participants (6.83 ± 0.94) compared to the male participants (9.00 ± 1.00; *p* < 0.001) (Figure 2). There were no significant differences over time for either the objective or subjective measures in the male participants.

### 4.1 Objective measures

The objective measures of aerobic performance did not significantly differ across the menstrual phases that were examined, which is in agreement with the results of previous studies of untrained, active, and trained female participants (Dombovy et al., 1987; Bemben et al., 1995; Smekal et al., 2007). The high test–retest reliability observed in the present study show that the objective aerobic parameters (VO_2max_, RER, peak blood lactate, and peak power) are not affected by the menstrual cycle in the female participants or tests performed at similar time intervals in the male participants.

### 4.2 Subjective measures

The major findings from this study would suggest that during menses, the female participants felt as if they were exerting more effort during the maximal graded exercise test and felt less recovered 24 h following the maximal aerobic exercise bout. These findings are in contrast with those of some previous research studies, which found no changes in RPE across the menstrual cycle phases (Bailey et al., 2000; Lara et al., 2020). This may be due to differences in the training status of the individuals in the present study compared to the previous studies. VO_2peak_ for the participants in the present study was ∼30 mL/kg/min compared to ∼50 mL/kg/min (Bailey et al., 2000) and ∼48 mL/kg/min (Lara et al., 2020). Individuals with low fitness or sedentary individuals have been shown to perceive greater exertion during exercise compared to trained individuals (Travlos and Marisi, 1996; Chen et al., 2002). Endurance training enhances oxidative enzyme activity and mitochondrial content, which results in a different metabolic profile compared with other types of training (Egan and Zierath, 2013; Al-Khelaifi et al., 2018). This could impact how female athletes perceive effort compared to untrained female participants. In addition, elite and high-level athletes have been shown to have increased pain tolerance, higher pain thresholds, and lower pain intensity (Pettersen et al., 2020). In addition, endurance athletes have been shown to have better pain tolerance and lower pain intensity than non-athletes (Pettersen et al., 2020).

On the other hand, the present findings are analogous to results found in other studies, which had individuals with lower reported physical activity levels (Caldwell Hooper et al., 2011). One study measured RPE and perceived pain during a treadmill exercise protocol at 65% of measured VO_2max_ in regularly menstruating female participants who participated in low levels of physical activity (Caldwell Hooper et al., 2011). The authors found that RPE and perceived pain ratings were higher in the female participants during the EF phase when compared to the late follicular and luteal phases. In addition, this study found a moderately strong positive correlation (r = 0.548) between perceived pain and RPE (Caldwell Hooper et al., 2011). This could be due to the fact that the female participants were shown to have increased clinical and experimental pain during the EF phase, which is associated with the declining levels of estrogen and progesterone (Martin, 2009). The authors concluded that RPE and perceived pain ratings were higher in the female participants during the EF phase, and this change is attributed to a drop in concentrations of both sex hormones, estradiol and progesterone, during this phase (Caldwell Hooper et al., 2011). However, this study did not measure hormone levels and used a calendar-based method for estimating the MC phase (Caldwell Hooper et al., 2011). Given the prevalence of menstrual cycle disturbances (Janse De Jonge et al., 2019), a calendar-based method would allow for the possibility that interindividual variations in hormone concentrations and hormone fluctuations across the MC could affect RPE.

There are a few possible explanations for the differences in subjective parameters across the menstrual cycle during exercise including pain perception, which in turn can influence RPE (Borg, 1982). Changes in mechanical and metabolic activities stimulate group III/II afferent neurons, which are involved in the perception of fatigue and pain (Jankowski et al., 2013; Pollak et al., 2014; Amann and Light, 2015). Increased concentrations of metabolites (ATP, lactate, and inorganic phosphate) during exercise have been shown to increase the perception of efforts during exercise (Silva et al., 2021; da Silva et al., 2019), which could be perceived as fatigue and/or pain. Given that the female participants achieved similar peak power, VO_2max_, RER, and blood lactate levels, it would be safe to assume that there was a similar level of mechanical and metabolic stimulation across the MC phases in the female participants. A possible explanation for the difference in RPE during exercise across the MC could be due to female participants having a higher sensitivity to a painful stimulus (lower pain threshold) during the follicular phase compared to other phases due to greater activation of brain areas related to general body awareness during the follicular phase (Veldhuijzen et al., 2013). This study found that the brain activity during a painful stimulus was found in the brain regions associated with general body awareness and motor skills and that areas associated with pain modulation were deactivated during the follicular phase (Veldhuijzen et al., 2013). A second possible explanation is the hypoactivity of the brain circuitry systems related to the inhibition of painful stimuli in the female participants. These systems have different activity levels throughout the menstrual cycle and operate more effectively around ovulation when estrogen is high and progesterone is low (Tousignant-Laflamme and Marchand, 2009). Previous research has shown that there is a correlation between pain and RPE during exercise (Rejeski, 1981). Our findings support the complicated interaction of physiological elements to perceive noxious stimuli and perception of efforts across the menstrual cycle.

Lastly, the female participants reported significantly lower PRS (recovery) values during EF compared to both O and ML. Previous research has shown that 24 h post-endurance exercise biomarkers (creatine kinase and IL-6) were higher during the mid-follicular phase than during the luteal phase (Hackney et al., 2019). Considering the antioxidant aspects and reduced levels of estrogen during the follicular phase (Tiidus, 2000), the biomarker results (Hackney et al., 2019), in addition to the findings from the present study, could indicate that there may be a need for greater recovery time in female participants following intense exercise in the early and mid-follicular phases. In trained individuals, greater sleep disturbance in the mid-luteal phase compared to the late-follicular phase was observed (De Martin Topranin et al., 2023). However, in another study, the female participants reported a lower quality of sleep during the 4 days of menses compared to mid-follicular and luteal phases (Baker and Driver, 2004). Decreased sleep quality may have impacted the recovery of female participants, following exercise in the EF phase.

#### 4.2.1 Limitations

The findings of the present study are generalizable only to the young, untrained, and healthy male population and young, healthy, naturally menstruating, and untrained female population. The present study did not directly measure hormone concentrations to test for the presence of corpus luteum and therefore cannot confirm the presence of ovulation in this group of participants. In addition, since hormone levels were not tracked, we could not confirm adequate progesterone levels in the ML phase. Both of these considerations are necessary because data from individuals with disturbed MCs, with smaller hormone fluctuations, could have been included in the data analysis. Subtle differences in the menstrual cycle could affect performance and perception of performance (Janse De Jonge et al., 2019). It was assumed that ovulation could accurately be determined by a spike in BBT. It has been articulated that BBT tracking may not be the most reliable method for tracking MC and estimating ovulation (Guermandi et al., 2001); however, the cost and practicality of this method reinforced its selection of use to schedule the visit during the ovulatory phase in this study (Guermandi et al., 2001).

The measurement of RPE is influenced by factors such as sex, age, fitness level, and familiarity with the testing equipment and protocol (Haddad et al., 2017). Psychological factors possibly including leadership, psychological resistance, and endurance level were previously found to have influences on RPE (Coquart et al., 2012). However, this study attempted to test for psychological impact using the POMS-B mood questionnaire as a screening tool. There were no observed differences for any of the POMS-B categories across the menstrual cycle for the female participants or across time for the male participants.

#### 4.2.2 Conclusion

The results of the present study suggest that the objective measures of aerobic performance such as VO_2max_, HR_max_, RER, peak blood lactate, and maximal workload are not significantly influenced by menstrual cycle phases. The parameters measured in this study also did not significantly vary across the three timepoints for the male participants, indicating that maximal aerobic performance is relatively stable across 3–6 weeks in untrained participants who were instructed to maintain stable physical activity levels for the duration of the study.

Furthermore, the female participants reported higher RPE during GXT and lower perceived recovery following GXT in EF compared to O and ML. This could be due to changes in the brain activity that inhibit pain perception (Tousignant-Laflamme and Marchand, 2009), a dramatic drop in estradiol and progesterone, an interaction of each of these influences, or something unknown. Present findings warrant a more in-depth analysis of possible neural and hormonal contributors to exercise performance and tolerance across the menstrual cycle, especially during menses.

The findings of the present study suggest controlling for the MC phase in exercise research and training. Physical discomfort for female participants may be higher during menses; therefore, it is recommended to track, account for, and schedule visits based on the MC phase if perceived exertion and recovery are important outcomes.

## Data Availability

The raw data supporting the conclusion of this article will be made available by the authors, without undue reservation.
